# Implementing a Food Prescription Program during COVID-19: Benefits and Barriers

**DOI:** 10.3390/healthcare12020182

**Published:** 2024-01-12

**Authors:** David Himmelgreen, Nancy Romero-Daza, William Alex Webb, Jacquelyn N. Heuer, Deven Gray, Gabrielle R. Lehigh

**Affiliations:** 1 Department of Anthropology, University of South Florida, Tampa, FL 33620, USA; daza@usf.edu (N.R.-D.); williamwebb@usf.edu (W.A.W.); heuerj@usf.edu (J.N.H.); devengray@usf.edu (D.G.); glehigh@usf.edu (G.R.L.); 2 Center for the Advancement of Food Security and Healthy Communities, University of South Florida, Tampa, FL 33620, USA

**Keywords:** food insecurity, food prescription program, diet-related chronic diseases, diabetes, hypertension, body mass index (BMI), health clinic, food bank, health outcomes, COVID-19

## Abstract

Food prescription programs (Food Rx) have the potential to improve management of diet-related chronic diseases or underlying conditions such as type 2 diabetes (T2D), hypertension, and high body mass index (BMI) among food-insecure patients. The purpose of this study was to examine the effectiveness of a Food Rx program implemented in two community-based clinics in Florida. Data were collected through researcher-administered surveys (food insecurity, demographics, and socio-economic variables) and biometric data (HbA1c, blood pressure, and BMI). Key results include the following: (1) Hispanic patients are more likely to utilize the program than their Black and White counterparts (*p* < 0.001); (2) older patients (≥50 years) have a higher food redemption rate when compared to younger patients (36–49.9 years); (3) food redemption rate is negatively associated with food security scores indicating improvements in food security status over time (r^2^ = −0.184, *p* = 0.036); (4) diabetic patients with higher baseline HbA1c (>9%) have significant reductions in glycated hemoglobin (*p* = 0.011) over time as compared to patients with lower baseline values (<7%); and (5) patients enrolled in the program for at least 6 months have a significant reduction in systolic blood pressure (*p* = 0.051). Changes in BMI were not significantly associated with redemption rates. This study is significant as it offers insights into the potential benefits and challenges of implementing Food Rx programs to address diet-related chronic diseases among underserved populations.

## 1. Introduction

Food insecurity (FI), the lack of availability or access to safe, nutritionally adequate, and socially acceptable food, is a longstanding problem in the United States (U.S). Early during the COVID-19 pandemic (2020), FI increased to 14.8% among all households with children and to 10.5% among all households [1]; the highest levels since the Great Recession of 2008–2009. The effects of FI can be far-ranging: chronic hunger, macro- and micronutrient deficiencies, and increased risk of infectious diseases. Similarly, FI is associated with the consumption of energy-dense and nutritionally unbalanced food, which can lead to diet-related chronic diseases (DRCDs) such as type 2 diabetes (T2D), cardiovascular disease (CVD), and hypertension [2]. Moreover, food-insecure individuals can experience physical, psychological, and economic burdens exacerbating their health risks [3]. While programs like the federal Supplemental Nutrition Assistance Program (SNAP) and local food banks and pantries ameliorate these issues in the short term, they do not typically address the longer-term prevention and management of FI-associated DRCDs [4].

Recently, food prescription (Food Rx) programs, also known as food as medicine, have been implemented in different settings [5,6,7,8,9]. Food Rx programs have been shown to improve food security status among vulnerable populations whose nutritional health is compromised [10,11]. For example, these programs have been shown to increase the intake of healthy foods such as fruits and vegetables [12], while also providing education on dietary management, food preparation, and food storage [13]. Interestingly, several Food Rx programs began during the COVID-19 pandemic when food assistance services had to make significant changes in a short period of time in response to social distancing practices and guidelines [14].

As COVID-19 rapidly spread during the second half of 2020, an estimated 3.4 million people were food-insecure in Florida including one in four children [15]. As expected, there were marked differences in FI by race and ethnicity, with Blacks and Hispanics more likely to be experiencing FI as compared to their White counterparts [15]. Other data show that 10.6% of the state’s population was food-insecure with rates ranging from 8.5% to 16.5%, depending on the county [16]. The prevalence of FI in Pinellas County, where this study took place, was 12.2% in 2020.

Early during COVID-19, the demand for food assistance rose dramatically in Tampa Bay. For example, prior to the pandemic, Feeding Tampa Bay (FTB) provided five to six million meals per month; this number nearly doubled after the start of the pandemic [17]. To meet the demand and execute social distancing practices and guidelines, FTBB and its affiliate agencies ramped up their services, for instance, initiating mega drive-through food pantries that served several hundreds of families at a time. In the midst of this period, the Food Rx was set to begin in March 2020 but was pushed back to September of that year.

The purpose of this study is to present results from a Food Rx program (heretofore, Food Rx) in Tampa Bay, Florida that began in September 2020 and ran through 2022. The Food Rx operated during the period when the full brunt of the COVID-19 pandemic was experienced by the patient-participants, staff, and researchers.

## 2. Materials and Methods

This Food Rx reflects a collaboration between Feeding Tampa Bay (FTB), Evara Health (EH), and the Center for the Advancement of Food Security and Healthy Communities (CAFSHC) at the University of South Florida (USF). The Food Rx was funded by a grant from the Humana Foundation. The study presented here was approved by the USF IRB (000451). FTB, a part of the Feeding America Network, provides food and services to food-insecure individuals and families living in 10 counties in west Central Florida. EH provides patient-centered health care at 11 clinic locations throughout Pinellas County, Florida. EH receives Health and Human Services funding and has Federal Public Health Service status. The CAFSHC addresses the role that FI has on the community and individual health through research and evaluation, program development, and education and training.

The Food Rx was implemented at two EH clinics serving primarily adult patients, many with diet-related chronic diseases/conditions (e.g., T2D, CVD, hypertension, high BMI). All patients that enrolled in the Food Rx received $25/week vouchers when they visited the clinic. To decrease the number of visits needed, clinic staff provided the vouchers as a set of four each month. Each voucher could be redeemed for food (e.g., fresh produce, canned fruits and vegetables, boxed grains, pre-packaged meal kits) either at the on-site food pantries at the clinics or at FTB’s “Grocery on the Go” (GoG) mobile food pantries. The GoG mobile pantries visited several locations weekly in areas where patients lived and worked. Instead of providing standard food packages, the Food Rx program emphasized client choice, allowing patients to choose their fresh produce and other food items based on their individual preferences. Mostly non-organic fruits and vegetables were available, with organic produce available occasionally. To assess the impact of the program, the CAFSHC collected quantitative data including food voucher redemption, biometric data collected by clinic staff, and food security surveys, as well as qualitative data through participant interviews and systematic observations. This study focuses on quantitative data, with qualitative results presented elsewhere (manuscript under review).

### 2.1. Timeline, Recruitment, and Data Collection

**Figure 1:**
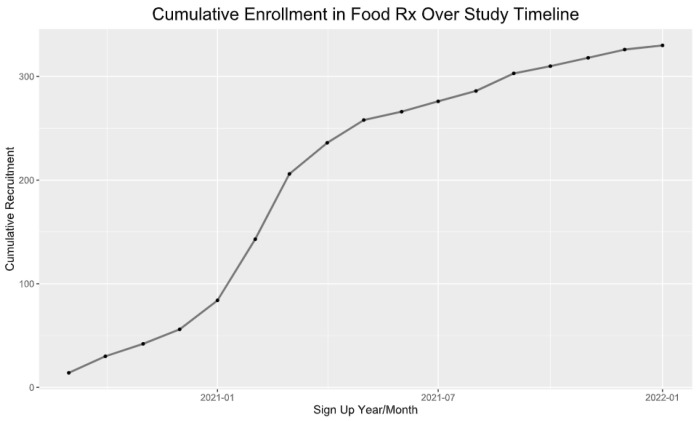
Recruitment was ongoing throughout the project, with a notable increase in enrollment at approximately the 6–8 months mark.

Using a modified two-question food security screening tool [18,19], clinic staff identified eligible adult patients (18+ years) to refer to the program. In total, a purposive convenience sample of 330 patients agreed to participate. Recruitment occurred on a rolling basis through the 18-month study period, with a sharp increase occurring 6–9 months after it began. Figure 1 shows the cumulative participant enrollment over the course of the study. Informed consent was obtained from each participant. Upon enrollment, participants agreed to complete the initial survey and to be contacted by phone to answer the follow-up surveys at three-month intervals. They were also informed that they could be invited to participate in an in-depth interview.

As part of routine care, EH staff collected various biometric data when patients presented at the clinic, including weight (in kg), height (in cm), BMI, hemoglobin A1c, and systolic and diastolic blood pressure (mm Hg). Initial plans to capture waist and hip circumferences were discarded early on due to feasibility constraints. EH clinic staff were responsible for collecting and inputting all the data related to biometrics and voucher redemptions.

Changes in food security were evaluated using a multi-component instrument composed of several previously validated surveys and administered by CAFSHC researchers at three timepoints for each participant: during program enrollment, at 3 to 6 months, and within 6 and 12 months. The survey captured sociodemographic information (such as self-reported gender, race/ethnicity, employment status, marital status, government assistance, etc.), and food security status via the United States Department of Agriculture’s 10-questions Household Food Security Survey Module (HFSSM). Additional data on dietary consumption including the 15-item Percentage Energy from Fat Screener (PFat) [20] and Food Frequency Questionnaire (FFQ) [21] focusing on the consumption of higher-fat foods will be reported elsewhere. This also applies to psychosocial measures of stress, social support, and loneliness collected through the 10-item Cohen’s Perceived Stress Scale [22]; the 12-item Multidimensional Scale of Perceived Social Support (MSPSS) [23,24]; and the 6-item De Jong Gierveld Loneliness Scale [25,26,27].

The HFSSM measures food security by generating raw scores which can be categorized into four levels: high, marginal, low, or very low food security, with lower scores indicating higher levels of food security and higher scores evidencing higher food insecurity. Of the 330 total Food Rx participants, 204 (62%) consented to completing the survey component over time. Along with food from the clinic and GoG food vouchers, participants received $10 Walmart gift cards to complete each of the first two surveys and a $25 gift card to complete the third survey. Despite these incentives however, survey completion rates declined substantially between the first, second, and third survey (see Table 1).

### 2.2. Data Analysis

Survey data (e.g., sociodemographic, food security) were collected in Qualtrics XM, while the clinical measures and food voucher redemption data were entered into Microsoft Excel 2021. These variables were combined into a master database to generate standard descriptive and inferential statistics using R v.4.0.4 and IBM SPSS v.26.

The analysis of quantitative data was two-fold: (1) an examination of differences in survey responses, redemption rates, and program participation across sociodemographic factors such as age, gender, race/ethnicity, income level, etc. and (2) an exploration of whether participation in the Food Rx (as measured by food voucher redemptions) predicts improvements in biometrics and food security status over time.

However, the impact of the Food Rx on biometrics was only appropriate to observe with participants experiencing outside-of-normal-range clinical measures. Consequently, sample sizes for each biometric, as well as survey responses through time (Table 1), varied. To make the most robust case, biometrics and FS scores were matched to total voucher redemptions within a one-month date range for analysis. To control for differences in the baseline scores, percent change was also used for comparisons. Additionally, the statistical tests used were dependent on the structure of the individual data subsets. They included paired t-tests, ANOVA, regression, and chi-square.

### 2.3. Challenges and Adjustments

The process of data collection was impacted by the COVID-19 pandemic. Early on during the evaluation period, the BFSRG team collected data from participants using Microsoft Teams by providing iPads to the participants while they were at the clinic to receive medical services. The team also made cold calls to administer the survey when participants did not complete it during their clinic visit. During Spring 2021, as the spread of COVID-19 slowed, the team started to collect data on-site at one of the clinics. However, because of social distancing guidelines and the inability to recruit potential participants in the waiting areas, this was impractical, and the team returned to collecting data virtually and calling participants to survey and interview.

## 3. Results

Two sets of results are reported here: (1) data from the total sample of participants enrolled in the Food Rx (*n* = 330); (2) data from those participants that completed one or more surveys throughout the program (*n* = 204).

### 3.1. Patient Attrition

Of the 330 patients that enrolled in the Food Rx, 60 (19%) withdrew from the program. These patients were enrolled for an average of 100 days (sd = 67.1; range 1–251 days) and redeemed food about two times (sd = 1–6). Withdrawals were identified during follow-up phone calls to patients absent from the clinic for more than two months. During these calls, clinic staff inquired about participants’ reasoning for withdrawing and, if amenable, offered accommodations allowing continued program participation. Only those who confirmed to staff that they would no longer like to participate were considered withdrawals. It was otherwise difficult to predict individual level of participation because some went weeks or months between redemptions. Participants’ main reasons for withdrawing from the Food Rx included: work demands and lack of transportation (43.3%), no longer being interested or the clinic not being able to contact patients (23.3%), no longer being food-insecure (17%), and issues unrelated to the program (18.3%). Approximately half (48.3%) of the participants who withdrew had taken at least one survey.

### 3.2. Underlying Diet-Related Chronic Diseases (DRCDs) or Conditions

All patients in the Food Rx were food-insecure, according to the two-question screener, and most reported at least one DRCD or condition (e.g., T2D, hypertension, high BMI). Nearly half (44%) of the patients had a high BMI (≥25). Hypertension affected 30% (*n* = 100) of patients, while 29% (*n* = 96) experienced type 2 diabetes (T2D). In total, 28 patients (8%) were managing two diet-related chronic conditions concurrently. Specifically looking at dual diagnoses: 2% (*n* = 8) had both hypertension and obesity, 3% (*n* = 10) had both hypertension and T2D, and an additional 3% (*n* = 10) had both T2D and a high BMI. A smaller subset of patients (2%, *n* = 6) had all three conditions: hypertension, T2D, and a high BMI.

### 3.3. Food Redemption

Participation in the Food Rx was measured by redemption rate i.e., the number of times patients redeemed food relative to the number of times they were prescribed, over the course of the program. The redemption rate was calculated using r = R/E.

Where r = redemption rate (proportion between 0 and 1); R = total vouchers redeemed; E = total vouchers received.

The mean number of food redemptions was 8.0 (sd = 7) for the total sample (*n* = 330) of patients in the Food Rx and 7.0 (sd = 7) for those that completed one or more surveys (*n* = 204). The average number of weeks enrolled in the program was 36 weeks (sd = 4) and 35 weeks (sd = 6) for each of these groups, respectively. Additionally, the food redemption rate for the total sample was 26% (sd = 22%) and 23% (sd = 22%) for the patients that completed at least one survey. However, it should be noted that the food redemption rate increased to 41% (sd = 23.51) for those patients that were actively enrolled in the Food Rx for at least three months.

**Figure 2:**
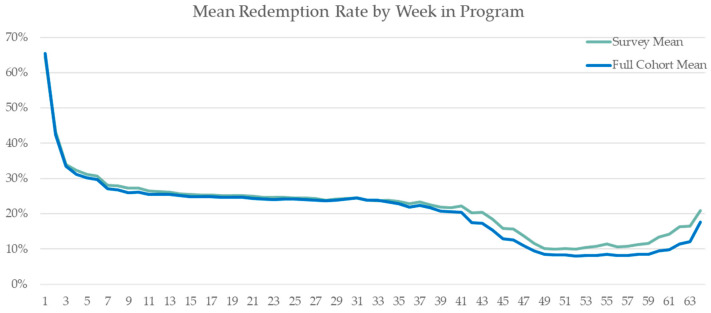
Mean participant redemption rate by week of enrollment.

Figure 2 displays the voucher redemption rates over time among the 303 Food Rx participants enrolled for varying lengths. To standardize comparisons, participants were ordered by weeks enrolled rather than calendar dates. So, the week 1 redemption rate reflects 303 participants since all were enrolled for at least 1 week upon enrollment. Subsequent weekly rates only include participants enrolled for at least that many weeks. For example, the week 3 rate reflects redemptions among participants active for ≥3 weeks. Since some patients signed up weeks after others, the sample size decreases in later weeks as fewer had longer enrollment periods.

Two lines show the redemption rates over the follow-up time. The total sample line depicts all 303 participants regardless of survey completion. The survey mean line shows only those who completed surveys. As seen, redemption rates declined at a near parallel rate over time regardless of survey completion.

### 3.4. Results from Patients Completing Surveys (n = 204)

Table 2 shows the sociodemographic for patients that completed one or more surveys during the program. The mean age of these patients was 57 y (sd = 12). Table 2 shows that nearly 78% were female, 45% were White, 24% were Black, and 23% were Hispanic. Additionally, 78.9% were unmarried, 18% were housing insecure, 58% earned less than $15,000 annually, and 78.9% received food assistance (e.g., SNAP, food pantries) in addition to Food Rx.

At the start of the Food Rx, 85% of these patients reported low or very low food security. Further, White patients were more likely to have very low food security (60.4%) when compared to their Hispanic (39.1%) and Black (35.4%) counterparts. A Pearson’s chi-square test was conducted to assess whether there was a relationship between racial/ethnic group and food security category after removing participants with high food security. After removing high food security participants, the results were statistically significant, χ^2^(6) = 14.176, *p* = 0.028. This indicates that there is an association between racial/ethnic group and food security category among participants with marginal, low, or very low food security. However, the results should still be interpreted with caution as more than 20% of cells may have expected counts of less than five, violating test assumptions.

Post hoc column proportion comparisons were conducted using the Bonferroni correction to evaluate which racial groups showed differences in marginal and very low food security categories. For marginal food security, the proportion for White participants was significantly higher compared to Black participants. For very low food security, the proportion for Black participants was significantly higher than for White participants. No other comparisons were statistically significant at the 0.05 level after adjusting for multiple testing.

Table 3 shows that the food redemption rate was higher among Hispanic patients (33.46%) compared to their White (23.51%) and Black counterparts (13.68%). Redemption rates followed a scale-free distribution with a strong positive skew. Consequently, a log10 transformation was conducted to meet assumptions of normality to test these differences (Kolmogorov–Smirnov, *p* = 0.012).

A one-way ANOVA found a significant effect of racial group on redemption rates [F(3,144) = 5.769, *p* < 0.001]. Post hoc LSD and Bonferroni comparisons showed that the mean redemption rate was significantly higher for Hispanic participants compared to Black participants (*p* = 0.001). Additionally, the mean rate for Black participants was significantly higher than both Other racial/ethnic (*p* = 0.042) and White participants (*p* = 0.007) based on the LSD test. However, after Bonferroni correction, only the difference between Black and Other racial/ethnic participants remained significant (*p* = 0.042). Redemption rates did not significantly differ between any other racial groups in post hoc testing. Overall, these results indicate racial disparities in food prescription redemption rates, with lower rates seen among White and Other minority groups compared to Hispanic and Black participants. Further investigation into factors influencing these racial differences is warranted.

**Figure 3:**
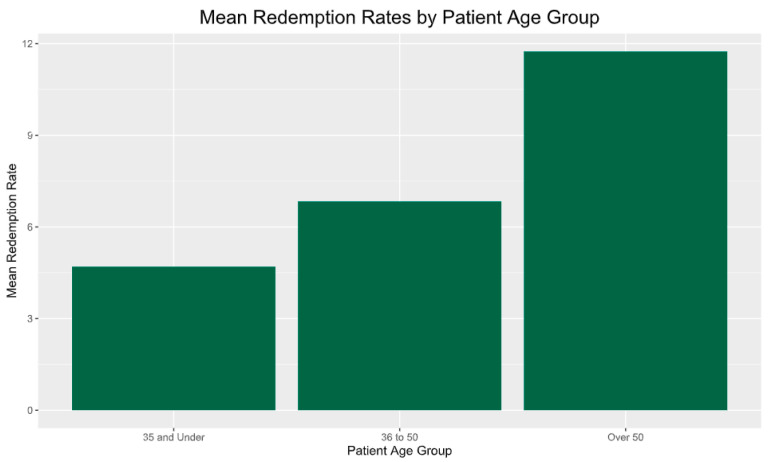
Mean participant redemption rates by age. Note: The ≥50-years age group has significantly higher redemption rates compared to the ≤35-years age group (*p* = 0.002) and the 36–49.99-years age group (*p* = 0.011).

Figure 3 shows that participants 50 y or older had a higher redemption rate (46.1%) than younger ones (25.7% for those 35 y and under). A second one-way ANOVA was conducted to determine if there were differences in redemption rates between age groups after applying a log10 transformation. There was a significant effect of age group on redemption rates at the *p* < 0.05 level for the three age groups [F(2,145) = 8.066, *p* < 0.001]. Post hoc LSD comparisons and Bonferroni comparisons indicated that the mean redemption rate for the over-50 age group was significantly higher than for both the under-35 age group (*p* = 0.002) and the 36–50 age group (*p* = 0.011). The under-35 and 36–50 groups did not significantly differ (*p* > 0.05). Overall, these results show that older adults (over 50) redeemed food prescriptions at a significantly higher rate than younger adults. Further research is needed to determine factors impacting redemption rate by age.

### 3.5. Changes in Food Security Status and Health Metrics during Food Rx

At baseline, 204 patients took the survey, 69 took the midpoint survey, and 41 completed the end-of-study survey. The surveys included the repeated HFSSM module to measure changes in food security status.

About half of the patients improved their food security score (lower raw number) and 30% improved their food security status (i.e., moved up at least one category, for example from very low to low food security, or from low to marginal food security) from baseline to the second survey. Of those patients completing a third survey, 44% improved their raw food security score and 24% improved their food security status. There was a significant negative correlation between food security score and food redemption rates between the first and second survey (r^2^ = −184, *p* = 0.036). In other words, a decrease in the food security score (i.e., improved food security status) was significantly associated with increased food redemptions.

### 3.6. Biometrics

Biometric measures (HbA1c, BMI, and blood pressure) were taken throughout the course of the Food Rx.

#### 3.6.1. Hemoglobin A1c (HbA1c)

The associations between voucher redemption rates and percent changes in HbA1c over time were modeled using multiple regression and ANOVA analysis, controlling for days between measurements, and initial HbA1c scores. There were 56 patients who had type 2 diabetes (T2D), had their HbA1c taken at least twice, and had participated in the program for at least three months. As the HbA1c measure is only collected quarterly, the analysis examined changes between the measurement closest to the patients’ enrollment date and the most recent collection. The mean baseline HbA1c was 8.43 (sd = 2.3). To meet normality assumptions, a single outlier (3.8 z-score) was removed (Kolmogorov–Smirnov, *p* = 0.200).

There was an average of 233 days (sd = 106) between HbA1c tests during which the mean percent change was −3.85% (sd = 20.21%). Patients experienced a range of changes with a minimum reduction of −58.46% and max increase of 73.13%. The mean voucher redemption rate for this period was 33.68% (sd = 30.13%). An initial multivariate regression model found that both unstandardized voucher redemptions (B = −0.048, *p* = 0.041) and baseline HbA1c (B = −0.375, *p* < 0.001) significantly predicted improvements on unstandardized HbA1c scores with a model power of 0.096.

**Figure 4:**
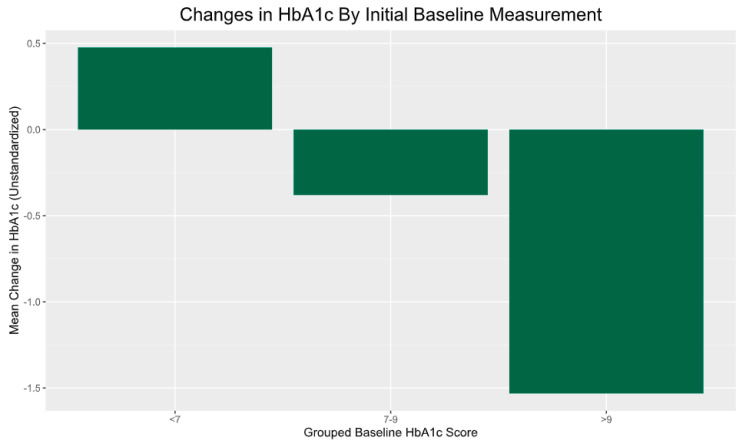
Food redemption rates and changes in HbA1c. Note: participants with higher baseline HbA1c (>9%) had significant reductions in HbA1c compared to their counterparts with lower HbA1c (<7%; *p* = 0.004).

To parse the influence of baseline HbA1c scores, participants were segmented into low (<7), moderate (7–9), and high (>9) baseline HbA1c groups. Analysis of the variance uncovered that the highest baselines (>9%) experienced significantly greater average reductions of −1.53% versus the low scoring group (0.477%), *p* = 0.004, suggesting regression toward the mean (Figure 4).

While a supplemental standardized multiple regression was not able to replicate the effects of voucher redemption, taken comprehensively, a general dose–response relationship seems present between voucher utilization and HbA1c improvements. However, the potential for change is also significantly related to initial blood sugar control status. If feasible, future research should consider segmenting groups based on HbA1c levels when evaluating the impact of Food Rx.

#### 3.6.2. BMI

Linear regression models were used to examine associations between voucher redemption rates and percent changes in BMI over the study period, controlling for days between BMI measurements, and initial BMI scores. There were 164 patients whose BMI at enrollment was ≥25, and who had participated in the program for at least 3 months. The range of baseline BMIs was broad with a mean of 35.55 (sd = 9.38). The values skewed slightly towards the low end with a few extremes on the high end. After calculating the percent change in BMI, this skewness persisted. Consequently, three outlier values were removed (+/− 3 z-score) to meet normality assumptions (Kolmogorov–Smirnov, *p* = 0.200).

There was an average of 248 days (sd = 88) between BMI measures during which the mean BMI change was −0.13% (sd = 4.57%). Patients experienced a range of BMI changes with a minimum of −17.44% and max of 13.98%. Mean redemption rate for this period was 25.96% (sd = 27.53%).

The overall model predicting percent BMI change was not significant [F(3,156) = 0.263, *p* = 0.852, R2 = 0.005]. Voucher redemption rates (B = 0.01, *p* = 0.443), days between BMI measurements (B = −0.00, *p* = 0.964) and baseline BMI (B = −0.019, *p* = 0.625), showed no significant relationships with percent BMI change. Non-significant findings persisted under different model approaches including using unstandardized BMI and redemptions numbers.

While the models showed non-significant relationships between voucher redemption rates and changes in BMI, at least two potential mitigating factors exist. First, redemption rates had very high variability across program participants. It remains plausible that food prescription programs could drive BMI improvements, given the higher and more sustained utilization rates. Additionally, the wide-starting BMI distribution could restrict the ability to detect changes, as the initial weight status may regulate the speed and degree of possible reduction. To control for this by using baseline BMI values as covariates, the research design and model may not have been sensitive enough to interpret those relationships.

#### 3.6.3. Hypertension

There were 77 patients with hypertension (systolic pressure 130+ mm/Hg; diastolic pressure above 80 mm/Hg) enrolled in the Food Rx for at least three months. Unstandardized values of both systolic (SBP) and diastolic pressure (DBP) were normally distributed (Kolmogorov–Smirnov, *p* = 0.2). On average, 265 days elapsed between measurements. Patients saw mean systolic decreases of 2.91 mmHg (sd = 17.73, range = −38 to +43 mmHg) and diastolic drops of 0.49 mmHg (sd = 10.61, range = −26 to +22 mmHg). The mean voucher redemption rate was 25.83% (sd = 26.55, range 0 to 96%).

A linear regression model predicting unstandardized systolic change was significant overall (F(3,73) = 6.53, *p* < 0.001, R2 = 0.212). However, redemption rates (B = −0.33, *p* = 0.076) and the time between biometrics (B = −0.027, *p* = 0.246) were non-significant, while baseline SBP strongly predicted the degree of change (B = −0.496, *p* < 0.001). A second model predicting unstandardized diastolic changes also achieved overall significance [F(3,73) = 5.67, *p* = 0.002, R2 = 0.189]. Consistently, baseline DBP values related significantly to changes (B = −0.624, *p* < 0.001), but redemptions (B = −0.11, *p* = 0.322) and the time between biometrics (B = −0.025, *p* = 0.083) did not. Overall, patients demonstrated small but measurable systolic and diastolic declines averaging 2.91 mmHg and 0.49 mmHg, respectively, meeting thresholds for clinical benefits in cardiovascular risk reduction.

**Figure 5:**
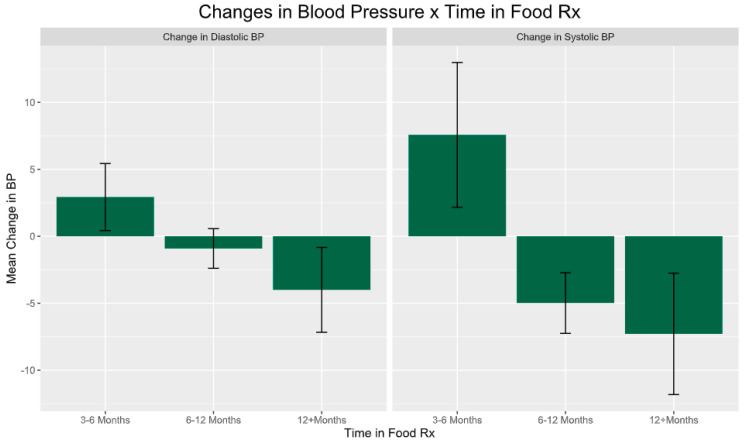
Changes in blood pressure over time for participants with hypertension. Note: participants enrolled for 3–6 months had increased SBP and a slight elevation in DPB while those enrolled for 6–12 months had a significant reduction in SBP (*p* = 0.051).

When stratifying participants by program exposure duration (e.g., 3–6 months, 6–12 months, 12+ months), an overall discrepancy emerged in systolic improvements [F(2,74) = 3.221, *p* = 0.046]. Notably, Figure 5 shows that those in the 3–6-month initiation phase experienced systolic increases up to +14.86 mmHg on average, significantly differing from the predominant subgroup of patients enrolled for 6–12 months who saw mean reductions around −12.55 mmHg (*p* = 0.051). Though the primary model did not identify effects related to the number of days in the program, probing change characteristics over binned time periods suggests an overall effect on time in the program that should further inform future evaluation and intervention strategies.

## 4. Discussion

While Food Rx programs have only been in existence for a short time, they have been shown to improve food security, increase fruit and vegetable intake, and provide knowledge and skills for the management of DRCDs [10,11,12]. Still, there is growing but limited data on the effectiveness of Food Rx programs on health outcomes [28,29,30]. This study contributes to filling this gap in the literature by examining the impact of one such program, which was implemented during COVID-19, to reduce food insecurity and its associated health consequences among patients presenting with chronic diseases. Frequently, the measure of program success comes through self-report of either increased fruit and vegetable consumption or patient satisfaction [31,32,33,34,35]. Specifically, the quantitative results reported herein attempt to extend these evaluations by isolating the impact of food redemptions as they relate to changes in food security and health.

The results suggest that, in general, the Food Rx program had a positive impact on the overall health and well-being of participants. The relationship between food voucher redemptions and improvements to food security was significant, if modest. As Schwartz (2020) [36] notes, the most immediate and obvious benefit is the provision of nutritionally rich foods. In this case, 330 BHS patients received such access, redeeming a total of 2144 vouchers. This increased access to food came at a time of great need, given the general social and economic instability experienced by individuals and families because of the COVID-19 pandemic.

However, measuring the impact of food voucher redemptions on biometric health, as a proxy for increases in healthy consumption of fruits and vegetables beyond self-report, was complex. As other recent studies have observed, reliably tracking improvements in metrics like HbA1c, BMI, and blood pressure depends heavily on patient behaviors towards both the Food Rx program itself and clinic adherence for testing. For instance, Xie et al. (2020) [37] found no significant effect of program utilization (food redemptions) on controlled HbA1c, whereas Bryce et al. (2017) [38] found program participation improved HbA1c levels for patients with uncontrolled diabetes. This is similar to the results of our study, wherein participants with Type 2 diabetes experienced an average 4% decline in HbA1c levels, which was significantly associated with redemptions but also greatly impacted by baseline glucose control. This finding is important, since lower HbA1c levels are critical in efforts to reduce the detrimental effects of Type 2 Diabetes. Further research should consider patient segmentation not only by DRCD but by status of condition as well.

The literature on the relationships between BMI and Food Rx are similarly mixed. For instance, while Bryce observed improvements in uncontrolled HbA1c, there was no such improvement with weight or blood pressure [38]. However, other studies have observed an improvement in BMI associated with increased fruit and vegetables intake from a Food Rx program [6,39]. While our study did not find a significant relationship between redemptions and BMI, there are several potential mitigating factors that warrant consideration. First, redemption rates varied greatly among participants in this study. It remains possible that higher, sustained utilization could drive BMI reductions. Second, participants had a broad-starting BMI distribution at enrollment. Initial weight status may impact the rate and magnitude of change achievable. Although baseline BMI was controlled for statistically, the research design may not have detected subtle interactions between starting BMI and voucher use. For instance, Hager et al., 2023 [6], had a substantially larger sample size. Although, it may also be that obesity is the consequence of multi-dimensional and contingent factors not easily addressed by modification of eating behavior [40].

The impact of Food Rx on blood pressure is slightly clearer with multiple studies generally finding improvements to both diastolic and systolic blood pressure among participants [39,40]. This was mirrored by our study which showed clinically meaningful reductions in blood pressure for the small subset of participants experiencing hypertension, although they were not significantly associated with voucher redemptions. Further, when evaluating changes over different program exposure periods, a discrepancy emerged. Participants in the first 3–6 months saw slight systolic increases, significantly different from those enrolled for 6–12 months who experienced mean systolic drops of 12.55 mmHg. This hints at a potential overall relationship between length of participation and blood pressure improvement not fully captured by our statistical models. Moving forward, these complex findings suggest that food prescription programs may confer cardiovascular benefits, but the role of voucher use in driving outcomes remains unclear. Research should continue working to isolate utilization patterns and participation duration thresholds that optimize blood pressure reductions. Coupling nutritional food access with targeted lifestyle counseling and education around hypertension management could also strengthen future interventions.

However, despite the limited positive results that Food Rx has on the health of participants, it is clear that the impact of such a program could be much greater with increased rates of utilization. As we observed, low redemption and high withdrawal rates point to the need to address the factors that act as barriers for individuals to derive all the potential benefits of participation in the program. In the Food Rx evaluated here, participants could only access the clinic-based pantry during days and times when medical services were provided (thus excluding weekends and evenings). Likewise, the GoG mobile pantry—while more easily accessible in the neighborhoods where people live—also operated during these days/times. Following our evaluation, the Food Rx program implemented a home delivery option that was effective in addressing time constraints and lack of transportation. Nevertheless, there is a clear need to implement other programmatic changes including the expansion of the days and hours of operation of the program, ideally to include evenings and weekends to accommodate individuals who prefer to redeem their vouchers in person. Likewise, the fact that 23% of the participants who withdrew from the Food Rx stated they were no longer interested in the program, suggests the need to increase engagement with patients to better understand the factors that affect participation and to tailor programs to the specific needs/preferences of the intended users.

The results suggest the need for further research to better understand the impact of the Food Rx program on clinical outcomes, to increase overall redemption rates, and to decrease attrition. Additional research is needed as well as to explore the factors that account for the observed differences in utilization of the program (as reflected in voucher redemption rates) by older participants and self-identified Hispanic participants as compared to other racial/ethnic and age groups. Here, the use of qualitative approaches could be especially useful to ascertain whether cultural/generational factors play a role in the patterns observed, given that all participants shared similar socioeconomic and food security backgrounds.

While more research is needed, there are things that could be done to address attrition and the rapid decline in food redemption over time. For example, an automated system could be developed to track food voucher redemptions and send out reminders to participants about picking up food at clinics and Groceries on the Go. Moreover, Food Rx staff could follow-up with those participants to find out why they are not using the program and to discuss possible solutions. Finally, bus tokens/car-ride fares could be provided to participants that do not have access to transportation.

The most recent data from 2022 show that 44.2 million Americans (12.8%) live in food-insecure households, an increase of over 10 million from the previous year [41]. The increase in FI is attributed to inflation, especially the high cost of food and housing, in addition to the scaling back and elimination of government assistance programs following the pandemic. FI, or the lack of access to enough safe and nutritious food coupled with the high prevalence of obesity at over 37% in the US [42], makes it imperative to test innovative programs, such as Food Rx, to reduce the high health and economic costs of DRCDs.

Although the results from this Food Rx program offer promise, there are several limitations which include the following: (1) convenience sampling; (2) sampling bias resulting from only 62% of the Food Rx participants agreeing to completing the surveys; (3) participant attrition leading to a decline in sample size during follow-ups; and (4) a relatively small number of participants with DRCDs. These could have biased the results and limited their generalizability. Nonetheless, given the paucity on this topic, the findings from this study provide a glimpse into the potential benefits and challenges of Food Rx programs. We hope this study also generates questions that will stimulate further research on this promising intervention to address the impact of food insecurity on diet-related chronic diseases.

## 5. Conclusions

Food Rx programs have been in existence for a short time and while few studies have been conducted, they do show promise in improving food security status, increasing fruit and vegetables intake, and providing knowledge and skills for the management of DRCDs. The results from this study show similar findings in addition to providing data on health outcomes. Most notably, higher rates of program utilization (food redemption rate) are associated with improvement in food security, improvements in HbA1c levels among participants with T2D, and small but measurable improvements in blood pressure. Further, Hispanic participants were more likely to redeem food than their Black and White counterparts and older participants had a higher redemption rate than younger ones. More research needs to be conducted to understand and address these racial and age differences. Although the study results are promising, caution must be taken in their generalizability. This Food Rx program was implemented during the height of the COVID-19 pandemic which presented many challenges including participant attrition, the small number of participants with DRCDs that completed the longitudinal study, and the difficulties in following up with participants that either dropped out of the program or who intermittently redeemed food. Much more research must be conducted to not only evaluate Food Rx programs but also to enhance their effectiveness.

## Figures and Tables

**Table 1:** Number of participants who took surveys throughout the study, including the average days between each survey.

	# of Surveys	Mean Days between Surveys	St. Dev. between Surveys
Survey 1	204	-	-
Survey 2	69	151.80	64.76
Survey 3	41	239.77	63.01

**Table 2:** Participant sociodemographics.

Socio-Demographics of Participants Who Completed Survey (*n* = 204)			
		Count	Column *n* %
What is your gender?	Female	160	78.4%
	Male	44	21.6%
What race do you identify as?	White	91	44.6%
	Black	48	23.5%
	Hispanic	46	22.5%
	Other	19	9.3%
Are you currently employed?	No	121	59.3%
	Yes	83	40.7%
What is the income level of your household?	$15,000 to $24,999	41	20.1%
	$25,000 to $34,999	25	12.3%
	$35,000 to $49,999	14	6.9%
	$50,000 to $74,999	3	1.5%
	Less than $15,000	115	56.4%
Do you currently receive other food assistance?	Yes	161	78.9%
	No	43	21.1%
What is your marital status?	Single	88	43.1%
	Married	43	21.1%
	Divorced	37	18.1%
	Separated	23	11.3%
	Widowed	13	6.4%
Which of the following best describes where you currently live?	Rent	114	55.9%
	Housing Insecure	37	18.0%
	Living with Family	11	5.5%
	Own	42	20.6%

**Table 3:** Food redemptions and enrollment in the Food RX by race/ethnicity.

Race/Ethnicity												
			White		Black		Hispanic		Other		Total	
			Count	%Total	Count	%Total	Count	%Total	Count	%Total	Count	%Total
Food Security Category		High	6	6.6%	3	6.3%	3	6.5%	3	15.8%	15	7.4%
		Marginal	5	5.5%	10	20.8%	9	19.6%	3	15.8%	27	13.2%
		Low	25	27.5%	18	37.5%	16	34.8%	5	26.3%	64	31.4%
		Very Low	55	60.4%	17	35.4%	18	39.1%	8	42.1%	98	48.0%
		Total	91	100.0%	48	100.0%	46	100.0%	19	100.0%	204	100.0%
**Race/Ethnicity**												
	**White**			**Black**			**Hispanic**			**Other**		
	**Mean**	**SD**	**Range**	**Mean**	**SD**	**Range**	**Mean**	**SD**	**Range**	**Mean**	**SD**	**Range**
Redemption Rate	23.51%	27.56%	127.27%	13.68%	17.44%	88.08%	33.46%	30.04%	97.39%	35.27%	29.87%	97.42%
Redemptions	8	10	48	5	7	38	13	11	32	15	13	39
Days in Program	270	92	424	274	88	331	281	63	300	288	74	301

## Data Availability

The data presented in this study are available on request from the corresponding author with permission from FTB.
